# Glucose starvation suppresses gastric cancer through targeting miR-216a-5p/Farnesyl-Diphosphate Farnesyltransferase 1 axis

**DOI:** 10.1186/s12935-021-02416-7

**Published:** 2021-12-25

**Authors:** Ruiyang Zhao, Bo Cao, Hanghang Li, Tian Li, Xingming Xu, Hao Cui, Huan Deng, Bo Wei

**Affiliations:** 1 Medical School of Chinese PLA, Beijing, China; 2 Department of General Surgery, First Medical Center, Chinese PLA General Hospital, Beijing, China; 3 School of Basic Medicine, Fourth Military Medical University, Xi’an, China

**Keywords:** Gastric cancer, FDFT1, Glucose starvation, miR-216a-5p, Glycolysis, Malignant progression

## Abstract

**Background:**

Fasting mimic diet is an effect approach for gastric cancer (GC) treatment. Exploring mechanisms of glucose deprivation-mediated GC suppression is required to develop novel therapeutic regimens. Farnesyltransferase 1 (FDFT1), as a novel target in basic research, has been reported to regulate malignant progression in some types of cancer. However, biological functions of FDFT1 in GC are still unclear. This study focused on biological functions of FDFT1 in GC and the association between glucose starvation (GS) and FDFT1.

**Methods:**

The data derived from the Kaplan–Meier Plotter database were collected to identify the relationship between survival time and FDFT1 expression levels of GC patients. Bioinformatic analysis was performed to explore the biological functions of FDFT1. The expression levels of targeted genes and microRNAs (miRNAs) were detected with immunohistochemistry, quantitative real-time PCR and western blot. Malignant behaviors were measured using cell counting, cell counting kit-8, 5-ethynyl-2-deoxyuridine, wound healing, invasion transwell assays in vitro and constructions of subcutaneous and lung-metastatic tumors in vivo. The glycolysis of GC cells was determined by a series of metabolites, including lactate acid, pyruvic acid, ATP production, rates of glucose uptake, extracellular acidification rate and oxygen consumption rate.

**Results:**

FDFT1 was downregulated in GC and negatively correlated with pathological T stage, pathological TNM stage and cancer differentiation. High expression of FDFT1 also indicated better prognosis of GC patients. FDFT1 upregulation attenuated proliferation, migration and invasion of GC. miR-216a-5p was identified as a critical suppressor of FDFT1 expression and miR-216a-5p/FDFT1 axis regulated malignant behaviors and glycolysis of GC cells. GS suppressed malignant behaviors of GC by targeting miR-216a-5p/FDFT1 axis both in vitro and in vivo.

**Conclusion:**

This study illustrated novel mechanisms by which GS effectively suppresses GC. FDFT1 may become a potential prognostic indicator and novel target of GC therapy.

**Supplementary Information:**

The online version contains supplementary material available at 10.1186/s12935-021-02416-7.

## Background

Gastric cancer (GC) is one of the most malignant tumors worldwide. There was more than 1,000,000 new cases of GC and 769,000 deaths in 2020. Despite the mild decrease in health burden caused by GC, it still ranks the fifth for incidence and fourth for cancer-associated mortality worldwide [1]. A great number of patients were diagnosed at advanced or late stages due to atypical symptoms and lack of access to early diagnosis. Surgery plus adjuvant chemotherapy serves as the mainstream of GC therapy. Recurrence after surgery and chemoresistance induced by long-term usage of drugs significantly attenuate its efficacies. In recent years, targeted drugs have been available and proved as effective approaches for GC treatment [2, 3]. However, the insensitivity for subpopulation and high cost greatly hinder the wide application of targeted therapy. It is urgent to identify the landscape of carcinogenesis and develop comprehensive regimens for GC treatment.

Cancer cells require metabolic reprogramming and ectopic energy supply to adapt to the highly proliferative state [4]. Glucose is the main source of energy supply. Appropriate restriction of carbohydrate intake may function as a promising approach to assisting cancer treatment. Accumulating studies have shown that glucose starvation (GS) can impair malignant progression, chemoresistance and stemness of cancer [5–7]. Deprivation of glucose is also able to sensitize cancer cells to targeted drugs [8]. Clinical trials further verified the potential value of intermittent fasting diet for cancer patients [9, 10]. Nevertheless, GS treatment may have risks of cachexia and intolerance to radical treatment. How to mimic GS and make it more acceptable represent a hot topic of current research.

Farnesyl-diphosphate farnesyltransferase 1 (FDFT1) is an important enzyme that participates in the cholesterol biosynthesis and catalyzes the farnesyl diphosphate dimerization. Weng et al. showed that FDFT1 acted as a tumor suppressor and GS suppressed colorectal cancer by upregulating FDFT1 [11]. FDFT1 knockdown had the similar effects with GS in colorectal cancer and may become a promosing target of GS alternative methods. However, the oncological functions of FDFT1 remained controversial in some types of cancer [11–13] and the role of FDFT1 in GC has not been reported. It is eargerly required to identify its functions in GC development. The development of related basic research may contribute to the progress of clinical diagnosis and therapy.

Another scientific question is that mechanisms by which GS increases FDFT1 expression have not been elucidated by existing studies. microRNAs (miRNAs) belong to one type of non-coding RNAs and act as critical roles in many physiological and pathological processes. miRNAs can directly bind to mRNA 3’ untranslated regions (3’UTRs), downregulating the expression levels of targeted genes at the post-transcriptional level. Numerous studies reported that miRNAs could regulate malignant progression and interference of miRNA expression serves as an effective method for cancer treatment [14, 15]. The regulatory relationships between GS and miRNAs have been prelimanrily revealed [16]. Based on the evidence, it has been speculated that GS upregulates FDFT1 expression by affecting miRNA networks. In this study, we aimed to investigate the functions of FDFT1 in GC progression and mechanistic associations between GS and FDFT1 through regulating specific miRNAs, which might provide strong proofs for the development of GS treatment.

## Methods

### Clinical specimen

50 GC patients who received radical gastrectomy for gastric adenocarcinoma at Chinese PLA General Hospital from May 2019 to March 2020 were enrolled in this study. The GC tissues and the corresponding adjacent normal tissues were immediately harvested when the tumors were separated. Patients who underwent radiotherapy or chemotherapy before gastrectomy were excluded. The collection of clinical samples was approved by the Ethics Committee of Chinese PLA General Hospital. The informed consent from all the included patients has been obtained.

### Correlation and enrichment analysis of FDFT1

The GC genomic expression levels in The Cancer Genome Atlas (TCGA) database were downloaded. The top 300 genes with the most positive correlations with FDFT1 expression and the top 300 genes with the most negative correlations with FDFT1 expression were selected out using Pearson correlation analysis. The 600 genes were enriched for Gene Ontology (GO) and Kyoto Encyclopedia of Genes and Genomes (KEGG) analysis.

### Cell culture

The human GC cell lines (HGC-27, MGC-803, AGS, BGC-823, SGC-7901, MKN-28) and a human stomach epithelial cell line GES-1 were purchased from the American Type Culture Collection (Manassas, VA, USA). HEK-293 T, a human embryonic kidney cell line, was gifted from Wound Healing Laboratory, Chinese PLA General Hospital. The MGC-803 cell line labeled with luciferase (luc-MGC-803) was previously generated and stored in our laboratory. Cells were grown in the complete medium, which referred to the Dulbecco's modified Eagle's medium (DMEM, Gibco, Thermo Fisher Scientific, MA, USA) supplemented with 10% fetal bovine serum (FBS, Kang Yuan Bioscience, Tianjin, China) and 1% penicillin‑streptomycin (Gibco; Thermo Fisher Scientific, MA, USA). Cells were maintained at 37 °C and 5% CO_2_ atmosphere.

### Glucose starvation assay

To generate the normal glucose or GS condition in vitro, DMEM, no glucose (Gibco, Thermo Fisher Scientific) was supplemented with 10 mM or 2.5 mM glucose, respectively. Other types of substances and culture conditions were the same with complete medium [17]. To acclimatize cells to the conditions that were different from high-glucose DMEM, cells were cultivated in the normal glucose or GS condition for 48 h before the formal experiments.

### Cell transfection and lentiviral infection

Overexpression plasmids, short-hairpin RNAs (shRNAs) and miRNA mimics were synthesized by JTSBIO Scientific (Wuhan, China). Transfection of plasmids and miRNA mimics into cells was conducted using Lipofectamine 2000 (Invitrogen, USA) according to the manufacture’s protocol. Lentiviruses were produced with the Lenti-Pac HIV Expression Packaging Kit (GeneCopoeia, USA). The stably infected cells were selected by 5 mg/mL puromycin (Yuanye, Shanghai, China) for 10 days.

### Quantitative real-time PCR (qRT-PCR)

Total RNA was extracted with TRIzol reagent (Servicebio, Wuhan, China). mRNA and miRNAs were reversely transcribed with ExScript RT-PCR Kit (TaKaRa, Japan). For miRNAs, specific oligo dT was designed for each kind of miRNAs according to the stem-loop method. The expression of miRNAs and FDFT1 was detected by qRT-PCR using Archimed X6 (Rocgene, Beijing, China) and SYBR Premix Ex Taq II (TaKaRa). qRT-PCR data were calculated by the 2^−ΔΔCt^ method. b-actin and U6 served as the reference genes for mRNA and miRNA expression, respectively. The sequences of oligo dT and primers are listed in Additional file 4: Table. S1.

### Western blot (WB) analysis

Cells were harvested and lysed using Radioimmunoprecipitation assay (RIPA) buffer to extract the total protein. The quantification of total protein was performed using BCA protein assay reagent (Thermo Fisher Scientific). Then, protein samples underwent sulfate–polyacrylamide gel electrophoresis (SDS-PAGE). The separated proteins were transferred onto polyvinylidene difluoride (PVDF) membranes (Millipore, MA, USA). The membrane block was performed with 5% skim milk for 1 h and incubated with antibodies against FDFT1 (dilution factor 1:1000, ab195046), b-actin (dilution factor 1:2000, ab6267) at 4 °C overnight and the secondary antibody (dilution factor 1:3000, ab205718) at 25 ℃ for 1 h. ECL Western Blotting Substrate (Solarbio) was prepared and carefully dripped on the membranes. 180 kDa Prestained Protein Marker (Vazyme, Nanjing, China) was used to label the protein positions on membranes. WB images were shown using the Tanon 4600SF Imaging System (Tanon, Beijing, China).

### Immunohistochemistry

Samples were embedded in paraffin for long-term storage. Tissue sections were prepared using a graded alcohol series. The slides were blocked using 0.3% hydrogen peroxide for 15 min and then boiled in the aminomethane-EDTA buffer (pH 8.0) for another 30 min. 10% normal goat serum was used to block non-target antigens for 20 min. Slides were incubated with the primary antibody overnight at 4 °C and horseradish peroxidase (Copenhagen, Denmark). The staining was visualized using Metal Enhanced DAB Substrate Kit (Solarbio) according to the manufacture’s protocol.

### Detection of luciferase reporter activities

The wildtype and mutant sequences of mRNA 3’UTR of FDFT1 were inserted into pGL4.0 luciferase reporter plasmids. MGC-803 and HGC-27 cells were cotransfected with empty vectors, wildtype plasmids or mutant plasmids and negative control or miR-216a-5p mimics, respectively. They were harvested and lysed after 24 h of incubation. Luciferase activities were determined with Dual-luciferase Reporter Assay System (Promega, WI, USA).

### Cell counting assay

1 × 10^4^ treated cells were seeded in the 24-well plates and incubated at 37 °C and 5% CO_2_. At the indicated time, cells were suspended in DMEM and densities were counted with an automated cell counter (Bio-Rad, Shanghai, China). The total cell numbers were calculated based on the densities and volumes.

### Cell Counting Kit-8 (CCK-8) assay

Cell proliferation was detected with CCK-8 reagent (Biorigin, Beijing, China). A total of 3 × 10^3^ treated cells from each group were seeded in 96-well plates. DMEM was used to prepare 10% CCK-8 working solution. The medium in wells was replaced with 100 μL of 10% CCK-8 solution. The plates were incubated in the cell incubator for 1.5 h. The absorbance at 450 nm was measured using the microplate reader (Biotek, VT, USA) at the indicated time.

### 5-ethynyl-2-deoxyuridine (EdU) assay

2 × 10^4^ treated cells were seeded in the 96-well plates. After 10 h, EdU assay was performed with Cell Proliferation EdU Image Kit (Abbkine, Wuhan, China) according to the manufacture’s protocol. Cell nuclei was stained with DAPI (Solarbio) to label the total cell number and location. The red and blue fluorescence was analyzed by an inverted fluorescence microscopy (Nikon, Japan).

### Wound healing assay

MGC-803 and HGC-27 cells were seeded in the 6-well plates to achieve 80%-90% density. Wounds were created by manual scraping in each well. Wound images were acquired under an inverted microscope (Carl Zeiss, Germany, Oberkochen). The cells were then incubated for 24 h and images were recorded again. The distances of travels towards the center were measured to evaluate migration capability of GC cells.

### Transwell assay

5 × 10^4^ treated cells were harvested and resuspended in 200 μL FBS-free DMEM. They were carefully dripped in the upper chamber. 600 μL 20% FBS diluted by DMEM was added into the lower chamber. Cells were then incubated for 24 h. They were fixed using 4% paraformaldehyde for 20 min and stained with 0.1% crystal violet. The invasion cells adhered on the lower chamber were counted under the inverted microscope.

### Glycolysis analysis

Glucose Uptake Colorimetric Assay Kit, ATP Colorimetric Assay Kit, Lactate Assay Kit II, Pyruvate Colorimetric Assay Kit (Biovision, CA, USA) were used to detect glycolysis of GC cells. The experiments were conducted according to the manufacturer’s protocols, respectively. The absorbances at the indicated wave lengths were measured by the microplate reader.

### Extracellular acidification rate (ECAR) and oxygen consumption rate (OCR) assays

Cell glycolysis and oxidative phosphorylation were determined with Seahorse XF Glycolysis Stress Test Kit, Cell Mito Stress Test Kit and Seahorse Bioscience XF96 Extracellular Flux Analyzer (Agilent Technology, MA, USA). 5 × 10^4^ cells were seeded in 96-well cell culture XF microplates (Agilent Technology, MA, USA) and incubated for 10 h. ECAR and OCR levels were examined and analyzed by Seahorse Bioscience XF96 Extracellular Flux Analyzer. The drugs were sequentially added into wells of XF microplates as indicated.

### Animal experiment

Male 4-week BALB/c nude mice (Charles River, Beijing, China) were fed in a specific pathogen-free (SPF) environment. For the preparation of a xenograft model of subcutaneous GC, 5 × 10^6^ luc-MGC-803 cells were resuspended in serum-free DMEM and mixed with same volume of Matrigel. The mixture was subcutaneously injected into the mouse right flanks. The diets of NC group and fasting mimic diet (FMD) group were designed according to the study of Weng et al. [11]. The longest diameters (lengths) and shortest diameters (widths) of tumors were recorded every 7 days. Tumor volume = (length × width^2^) /2. For the investigation of GC metastasis, 2 × 10^6^ luc-MGC-803 cells were resuspended in phosphate buffered saline. They were then carefully injected into tail veins. After 30 days, tumors in the two models were imaged by In Vivo Imaging System (Perkin Elmer, MA, USA). Nude mice were euthanized by CO_2_ asphyxia and subcutaneous tumors were harvested for further determination of lactate acid concentration. This animal experiment was authorized by the ethics committee of Animal Center of Chinese PLA General Hospital.

### Statistical analysis

GraphPad Prism 7.0 (GraphPad Software, San Diego, CA, USA) and SPSS 25.0 (SPSS Inc., Chicago, IL, USA) were used for data analysis. Two-sided paired and unpaired t-test were used to compare the data difference after the normality of distribution was confirmed. All experiments in vitro were repeated independently three times. *P* value < 0.05 was regarded as significant difference.

## Results

### FDFT1 exhibits low expression and is positively correlated with prognosis of GC

**Fig.1:**
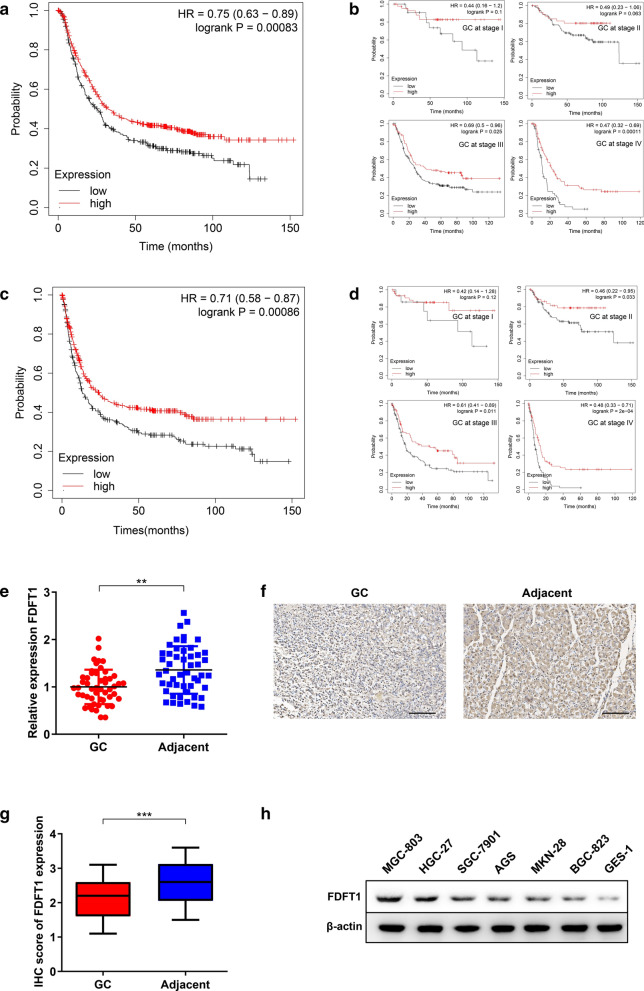
FDFT1 exhibits low expression and is positively correlated with prognosis of GC. **a** Data from Kaplan–Meier Plotter to show the OS between GC patients with low-expression and high-expression FDFT1. **b** Data from Kaplan–Meier Plotter to show the OS between GC patients as in (**a**), who were stratified according to TNM stage. **c** Data from Kaplan–Meier Plotter to show the PFS between GC patients with low-expression and high-expression FDFT1. **d** Data from Kaplan–Meier Plotter to show the PFS between GC patients as in (**c**), who were stratified according to TNM stage. **e** qRT-PCR analysis to show FDFT1 mRNA expression in 50 pairs of GC and adjacent noncancerous tissues. **f** IHC analysis to show FDFT1 protein expression as in (**e**). One case is displayed. Scale bar: 100 mm. **g** Histogram to display the IHC scores of clinical specimens as in (**e**). **h** WB analysis to determine FDFT1 expression in MGC-803, HGC-27, SGC-7901, AGS, MKN-28, BGC-823 and GES-1. Data are presented as means ± SD. **P < 0.01; ***P < 0.001

To explore the biological functions of FDFT1 in GC development, an online bioinformatic tool, the Kaplan–Meier Plotter, was employed to reveal the associations between prognosis and FDFT1 levels in GC tissues. Patients in the high-expression FDFT1 group had significantly better overall survival (OS) and progression-free survival (PFS, Fig. 1A, C). Next, the patients were stratified according to TNM stages. We found that the potential value of FDFT1 is increasing with the development of TNM stages. FDFT1 expression had a significant correlation with prognosis of patients, especially for patients at stages III and IV (Fig. 1B, D). The data suggested that FDFT1 had potential value of prognostic prediction and downregulation of FDFT1 expression might participate in GC progression.

Next, we collected 50 pairs of GC and matched normal tissues. qRT-PCR analysis indicated that FDFT1 exhibited lower expression in GC tissues (Fig. 1E). IHC examination was also used to detect FDFT1 protein expression of tissues in situ. FDFT1 exhibited lower expression in GC tissues compared to matched noncancerous tissues (Fig. 1F, G). We then analyzed the associations between FDFT1 protein expression and clinicopathological characteristics of included GC patients. The results showed that FDFT1 was negatively correlated with pathological T (pT) stage, pathological TNM (pTNM) stage and cancer differentiation. There was no significant difference of FDFT1 expression with age, gender, tumor size and tumor location of GC patients (Table 1). Otherwise, we chose six GC cell lines and one normal stomach epithelial cell line (GES-1) to examine FDFT1 expression. WB analysis showed that GES-1 had the highest FDFT1 protein levels. Otherwise, MGC-803 and HGC-27 cell lines had the highest FDFT1 expression among the six GC cell lines (Fig. 1H). Our data indicated that FDFT1 exhibited low expression in GC and might have potential in predicting the prognosis of patients.

**Table 1:** Correlation between FDFT1 expression and clinicopathological characteristics of 50 GC patients

Characteristics	Case number	High (n = 25)	Low (n = 25)	P value
Age at surgery (years)				0.569
< 60	28	15	13	
≥ 60	22	10	12	
Gender				0.529
Male	36	17	19	
Female	14	8	6	
pT stage				0.041
pT1+pT2	19	6	13	
pT3+pT4	31	19	12	
Tumor size (cm)				0.254
< 5	28	12	16	
≥ 5	22	13	9	
Location				1
Cardiac	20	10	10	
Non-cardiac	30	15	15	
pTNM stage				0.023
I + II	24	7	15	
III + IV	26	18	10	
Differentiation				0.034
Poorly	42	23	17	
Well	8	2	8	

*GC* gastric cancer, *FDFT1* Farnesyl-Diphosphate Farnesyltransferase 1, *pT stage* pathological T stage, *pTNM stage* pathological TNM stage

### Correlation and enrichment analysis of FDFT1

We employed bioinformatic analysis to preliminarily explore FDFT1 functions in GC. The 600 genes were identified, which included 300 genes that were most positively correlated with FDFT1 expression and 300 genes that were most negatively correlated with FDFT1 expression. The heatmap displayed the top 40 genes (Additional file 1: Fig. S1A). Top 10 KEGG pathways were enriched. Four categories are associated with malignant progression of cancer, including Cell adhension molecules, Tight junction, Gastric cancer and Focal adhension. Non-alcoholic fatty liver disease, Parkinson disease, Alzhemier disease and Huntington disease all belong to metabolic disorders [18, 19]. One carbon pool by folate participates in the regulation of nucleotide metabolism. PI3K-Akt signaling pathway is also a classical regulator of cell metabolism (Additional file 1: Fig. S1B). GO analysis is divided into three aspects, which includes biological process (BP), cellular component (CC) and molecular function (MF). Bubble charts displayed top 20 terms in each aspect. The majority of categories in the three aspects are related to cell adhension and membrane functions, which serve as the contributors of cancer progression [20]. FDFT1 dysregulation is also related with mitochondrial metabolism as shown by BP and CC (Additional file 1: Fig. S1C–E). Collectively, the results of bioinformatic analysis suggested the close association of FDFT1 with GC progression and metabolism.

### FDFT1 attenuates proliferation, migration and invasion of GC

**Fig.2:**
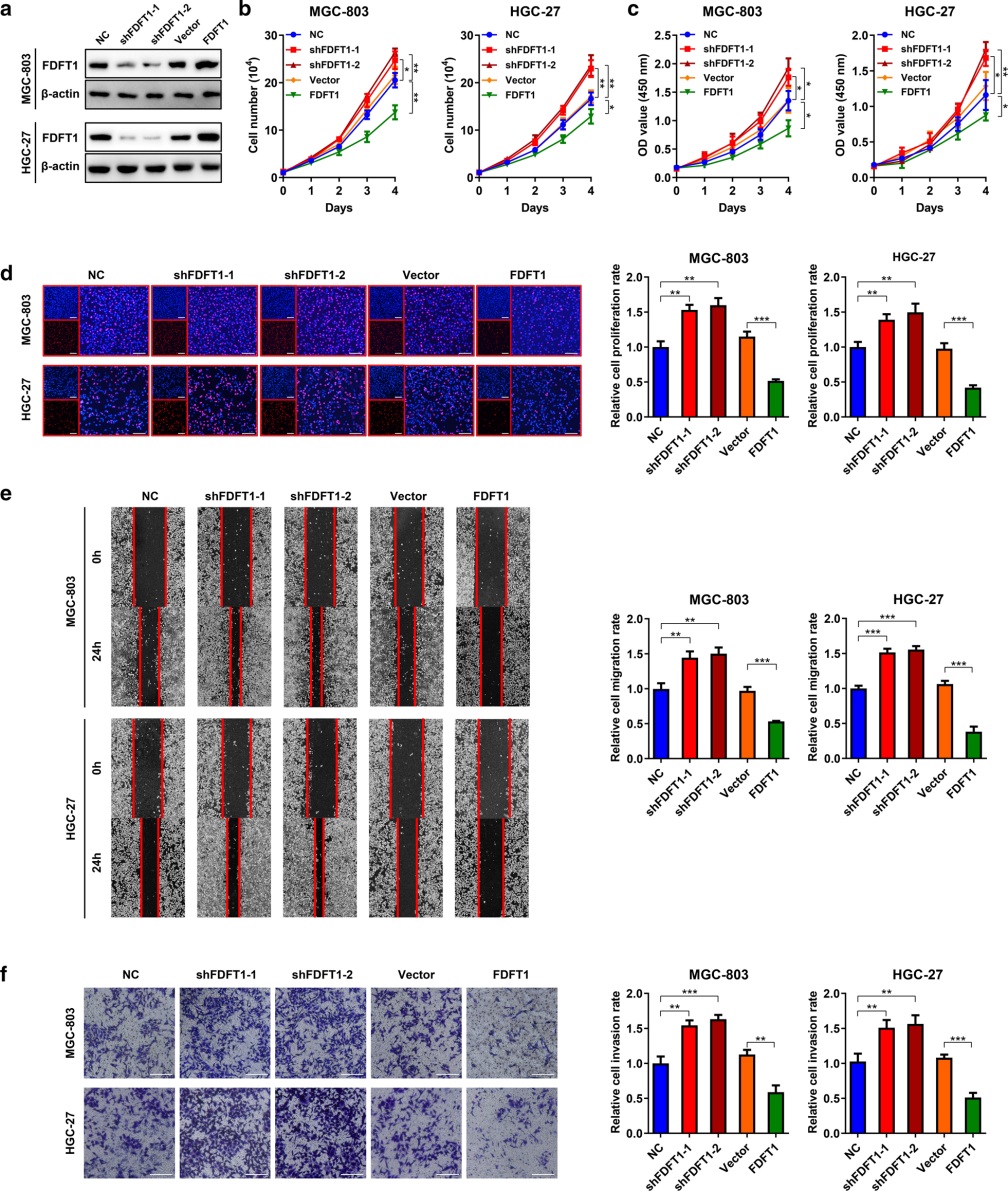
FDFT1 attenuates proliferation, migration and invasion of GC cells. **a** WB analysis to validate the expression of FDFT1 in MGC-803 and HGC-27 cells stably carrying lentivirus with NC shRNA, FDFT1 shRNA-1, FDFT1 shRNA-2, vector or FDFT1 overexpression plasmids, respectively. **b** Cell counting assay to show proliferation of MGC-803 and HGC-27 cells as in (**a**). **c** CCK-8 assay to show proliferation of MGC-803 and HGC-27 cells as in (**a**). **d** EdU assay to show proliferation of MGC-803 and HGC-27 cells as in (**a**). Histograms are on the right. Scale bar: 100 mm. **e** Wound healing assay to show migration of MGC-803 and HGC-27 cells as in **(a**). Histograms are on the right. **f** Transwell assay to show invasion of MGC-803 and HGC-27 cells as in (**a**). Histograms are on the right. Scale bar: 100 mm. Data are presented as means ± SD. *P < 0.05; **P < 0.01; ***P < 0.001

In consideration of FDFT1 expression levels in GC cell lines, we chose MGC-803 and HGC-27 cells for further experiments. GC cells stably carrying FDFT1 shRNAs or overexpression plasmids were constructed. WB confirmed their interference efficiencies (Fig. 2A). Cell counting, CCK-8 and EdU assays showed that knockdown of FDFT1 using shRNA-1 and shRNA-2 promoted GC cell proliferation. FDFT1 overexpression led to a significant proliferative suppression (Fig. 2B–D). Wound healing assay demonstrated that downregulation of FDFT1 expression also enhanced GC migration, while upregulation of it attenuated this capability (Fig. 2E). Transwell assay was conducted and validated that FDFT1 could inhibit cell invasion (Fig. 2F). Notably, two shRNAs both reduced FDFT1 expression and promoted GC malignant behaviors, which proved that there was no off-target effect of the two shRNAs on these malignant behaviors. Our findings demonstrated that FDFT1 acted as a tumor suppressor and upregulation of FDFT1 expression attenuated GC proliferation, migration and invasion.

### GS attenuates GC progression by upregulating FDFT1 expression

**Fig.3:**
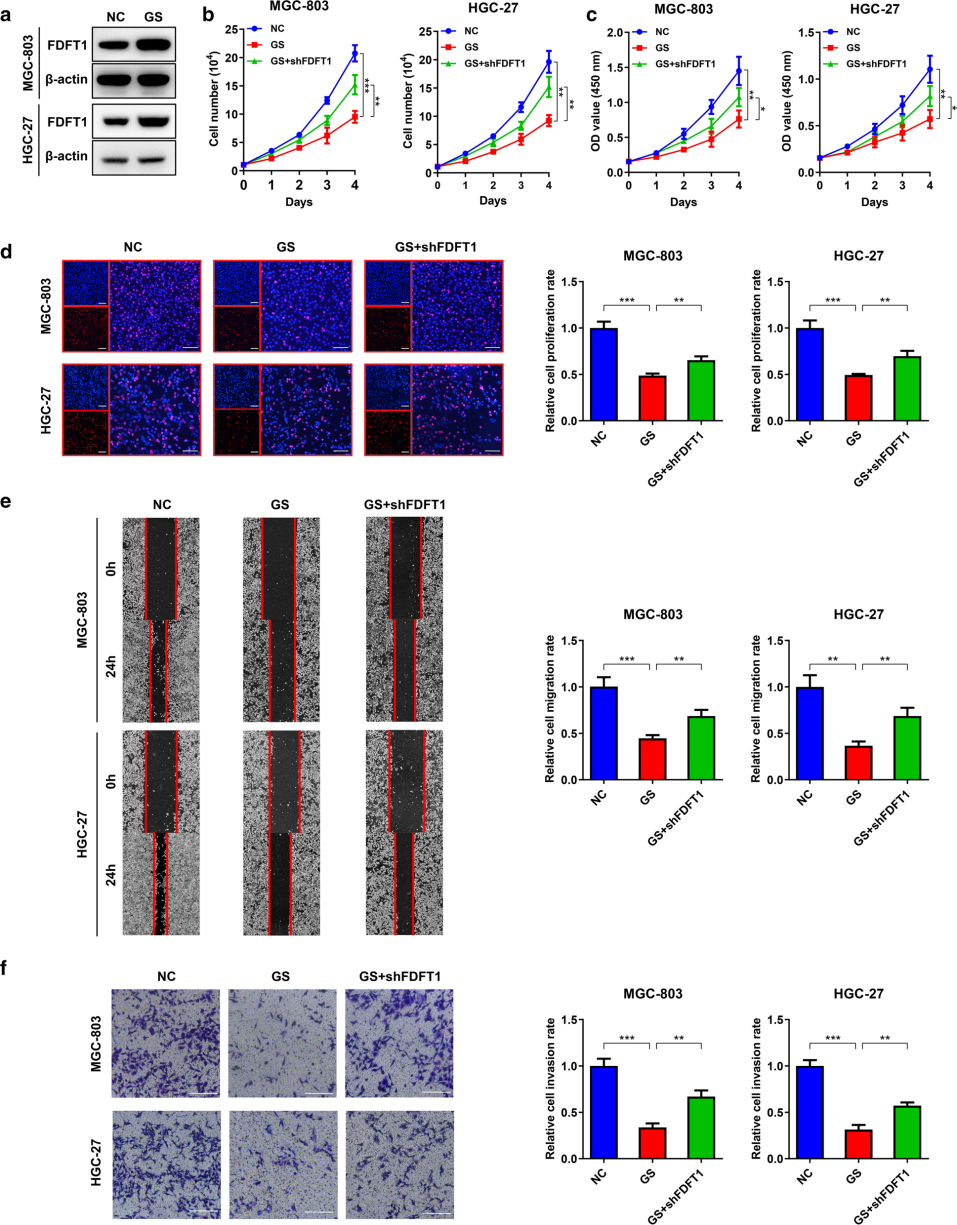
GS attenuates GC progression by upregulating FDFT1 expression. **a** WB analysis to show the expression of FDFT1 in MGC-803 and HGC-27 cells that were incubated under the normal glucose condition or the GS condition. **b** Cell counting assay to show proliferation of MGC-803 and HGC-27 cells under the normal glucose condition or GS condition, stably carrying lentivirus with NC shRNA or FDFT1 shRNA, respectively. **c** CCK-8 assay to show proliferation of MGC-803 and HGC-27 cells as in (**b**). **d** EdU assay to show proliferation of MGC-803 and HGC-27 cells as in (**b**). Histograms are on the right. Scale bar: 100 mm. **e** Wound healing assay to show migration of MGC-803 and HGC-27 cells as in (**b**). Histograms are on the right. **f** Transwell assay to show invasion of MGC-803 cells and HGC-27 cells as in (**b**). Histograms are on the right. Scale bar: 100 mm. Data are presented as means ± SD. *P < 0.05, **P < 0.01, ***P < 0.001

Many studies have proved the anti-cancer effects of glucose deprivation [5, 9]. Weng et al. reported that GS suppressed proliferation through elevation of FDFT1 expression [11]. Therefore, we first investigated the effects of GS on FDFT1 expression in GC. Consistent with the previous results, FDFT1 was upregulated in GC cells under glucose deprivation conditions (Fig. 3A). We then speculated that GS suppressed GC malignancy by increasing FDFT1 expression levels. Cell counting, CCK-8 and EdU assays indicated that GS impaired proliferative capability, which was significantly rescued by FDFT1 knockdown (Fig. 3B–D). Furthermore, wound healing and transwell assays also had the similar results (Fig. 3E, F). The data indicated that FDFT1 served as a mediator of glucose deprivation for tumor suppression.

### miR-216a-5p suppresses FDFT1 expression by binding to its mRNA 3’UTR

**Fig.4:**
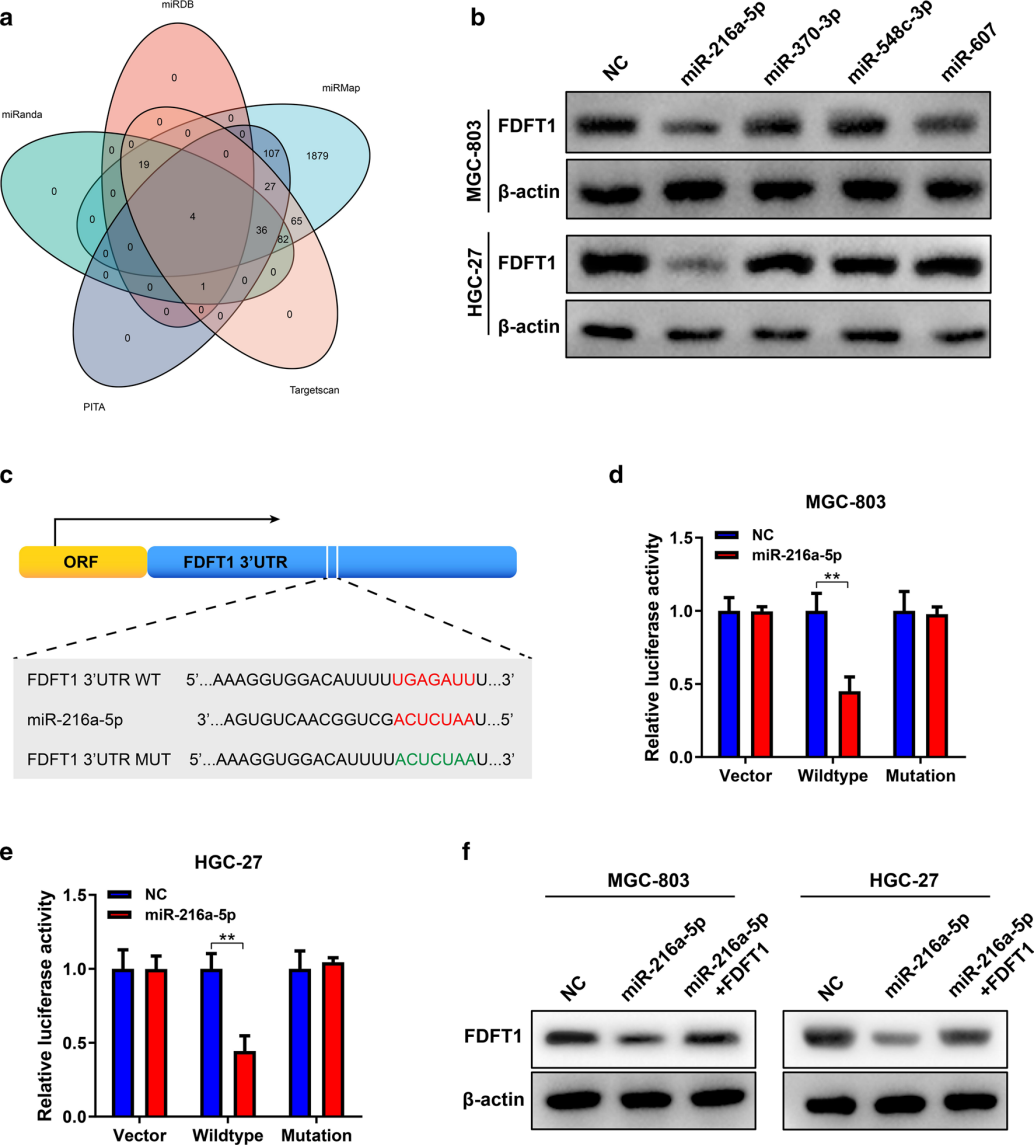
miR-216a-5p suppresses FDFT1 expression by binding to its mRNA 3’UTR. **a** Schematic illustration of miRNAs predicted by five online databases, including miRanda, miRDB, miRMap, PITA and Targetscan. **b** WB analysis to show FDFT1 expression in MGC-803 and HGC-27 cells transfected with NC mimics, miR-216a-5p mimics, miR-370-3p mimics, miR-548c-3p mimics and miR-607 mimics. **c** Schematic illustration of the wild-type (red) or mutated (green) sequences of the binding sites of miR-216a-5p and FDFT1 mRNA 3’UTR. **d, e** Luciferase reporter assay to investigate the relative activities in cell with co-transfected with empty plasmids, plasmids with the wildtype or mutated sequences of FDFT1 mRNA 3’UTR and NC mimics and miR-216a-5p mimics in MGC-803 (**d**) and HGC-27 cells (**e**), respectively. **f** WB analysis to show FDFT1 expression in MGC-803 and HGC-27 cells co-transfected with NC mimics or miR-216a-5p mimics and overexpression plasmids of FDFT1. Data are presented as means ± SD. **P < 0.01

miRNAs are a type of non-coding RNAs that regulate multiple physiological and pathological processes. A great number of miRNAs inhibit downstream mRNA expression by binding to their 3’UTRs. To elucidate miRNA networks against FDFT1 in GC, we used five bioinformatic tools to predict the potential miRNAs. As shown in the Venn diagram, four miRNAs were screened out by all these tools, which included miR-216a-5p, miR-370-3p, miR-548c-3p and miR-607 (Fig. 4A). Then the four miRNA mimics were synthesized and transfected into GC cells. qRT-PCR was conducted to verify their overexpression efficiencies (Additional file 2: Fig. S2A, B). Then, we found that only miR-216a-5p upregulation effectively suppressed FDFT1 expression (Fig. 4B). To confirm the binding sites of FDFT1 mRNA 3’UTR that were predicted by bioinformatic tools, we inserted wildtype and mutant sequences of FDFT1 mRNA 3’UTR into the luciferase reporter plasmids (Fig. 4C). miR-216a-5p overexpression significantly suppressed luciferase reporter activities. Nevertheless, miR-216a-5p could not reduce luciferase reporter activities of plasmids with mutant sequences (Fig. 4D, E). Otherwise, additional transfection of FDFT1 overexpression plasmids could rescue the miR-216a-5p effects on FDFT1 expression (Fig. 4F). The data proved that miR-216a-5p overexpression suppressed FDFT1 expression through binding its mRNA 3’UTR.

### miR-216a-5p/FDFT1 axis regulates GC malignancy and glycolysis

Since the relationship between FDFT1 and miR-216a-5p, experiments were used to testify the role of miR-216a-5p/FDFT1 axis. Elevation of miR-216a-5p levels facilitated GC proliferation, which was rescued by FDFT1 overexpression (Additional file 3: Fig. S3A–C). Then, we found that miR-216a-5p also potentiated migration and invasion. Additional upregulation of FDFT1 brought these malignant behaviors back to levels as the control group (Additional file 3: Fig. S3D, E).

**Fig.5:**
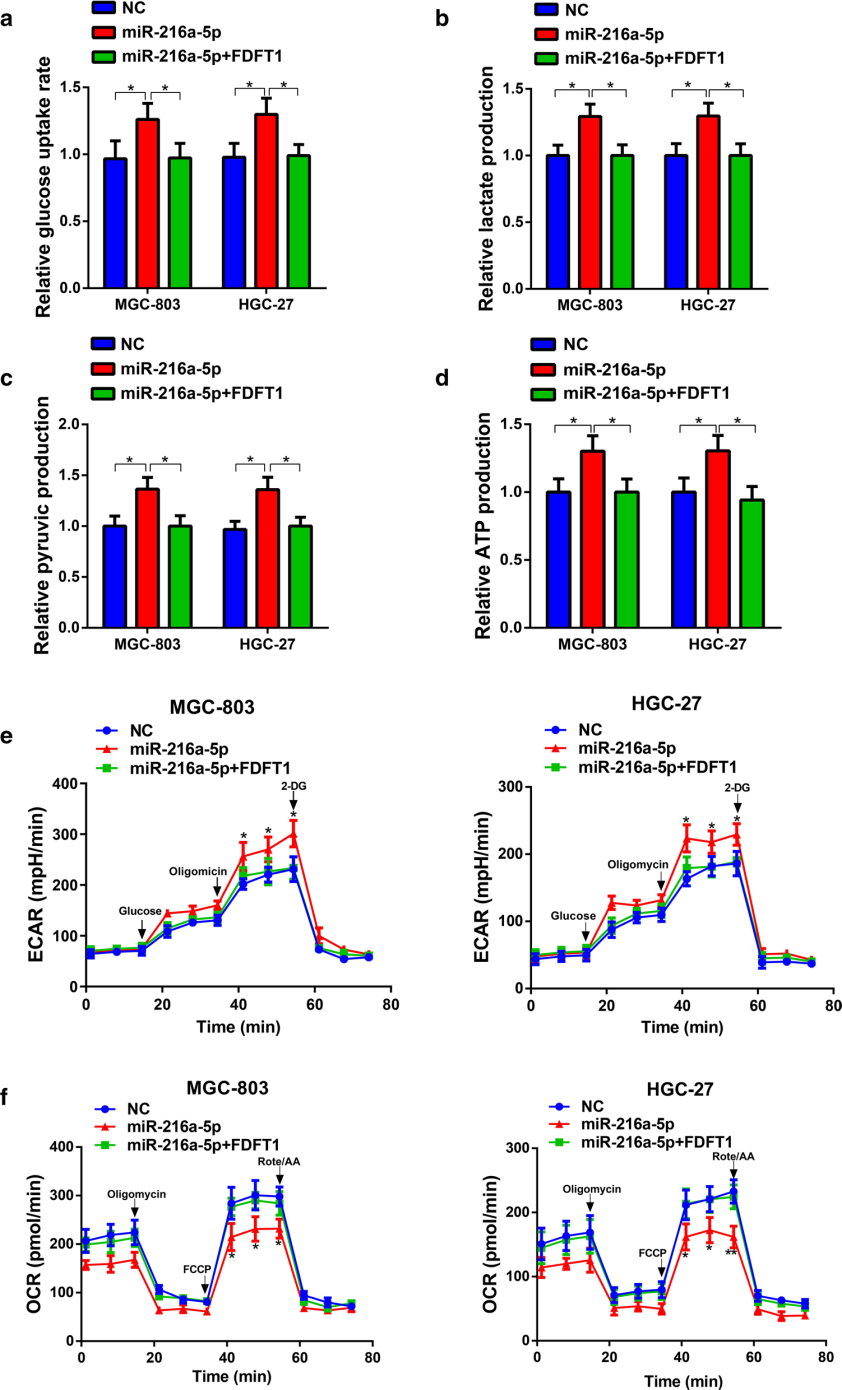
miR-216a-5p/FDFT1 axis regulates glycolysis in GC cells. **a-d** Glucose uptake (**a**) and the production of lactate acid (**b**), pyruvic acid (**c**) and ATP (**d**) were determined in MGC-803 and HGC-27 cells stably carrying lentivirus with vectors or FDFT1 overexpression plasmids and transfected with NC mimics and miR-216a-5p mimics, respectively. **e, f** ECAR (**e**) and OCR (**f**) assays of MGC -803 and HGC-27 cells transfected as in (**a-d**). A series of compounds were added at the indicated time. Data are presented as means ± SD. *P < 0.05, **P < 0.01

We then explored whether miR-216a-5p/FDFT1 axis regulates glycolysis of cancer. Glucose uptake serves as the initial process during the glucose metabolism. Lactate acid, pyruvic acid, ATP are the critical metabolites produced by glycolysis, and the levels of them reflect on glycolytic activities. Thus, we first chose these four indicators to measure cell glycolysis. Upregulation of miR-216a-5p enhanced lactate acid, pyruvic acid, ATP production and the speed of glucose uptake, which was rescued by FDFT1 overexpression (Fig. 5A–D). EACR and OCR are indicators of glycolysis and mitochondria activities, respectively. miR-216a-5p significantly enhanced ECAR while OCR was suppressed. Upregulation of FDFT1 expression could reverse this process (Fig. 5E, F). The data indicated that miR-216a-5p/FDFT1 axis could regulate GC malignant progression and glycolysis.

### miR-216a-5p serves as an important mediator of FDFT1 upregulation induced by glucose deprivation

**Fig.6:**
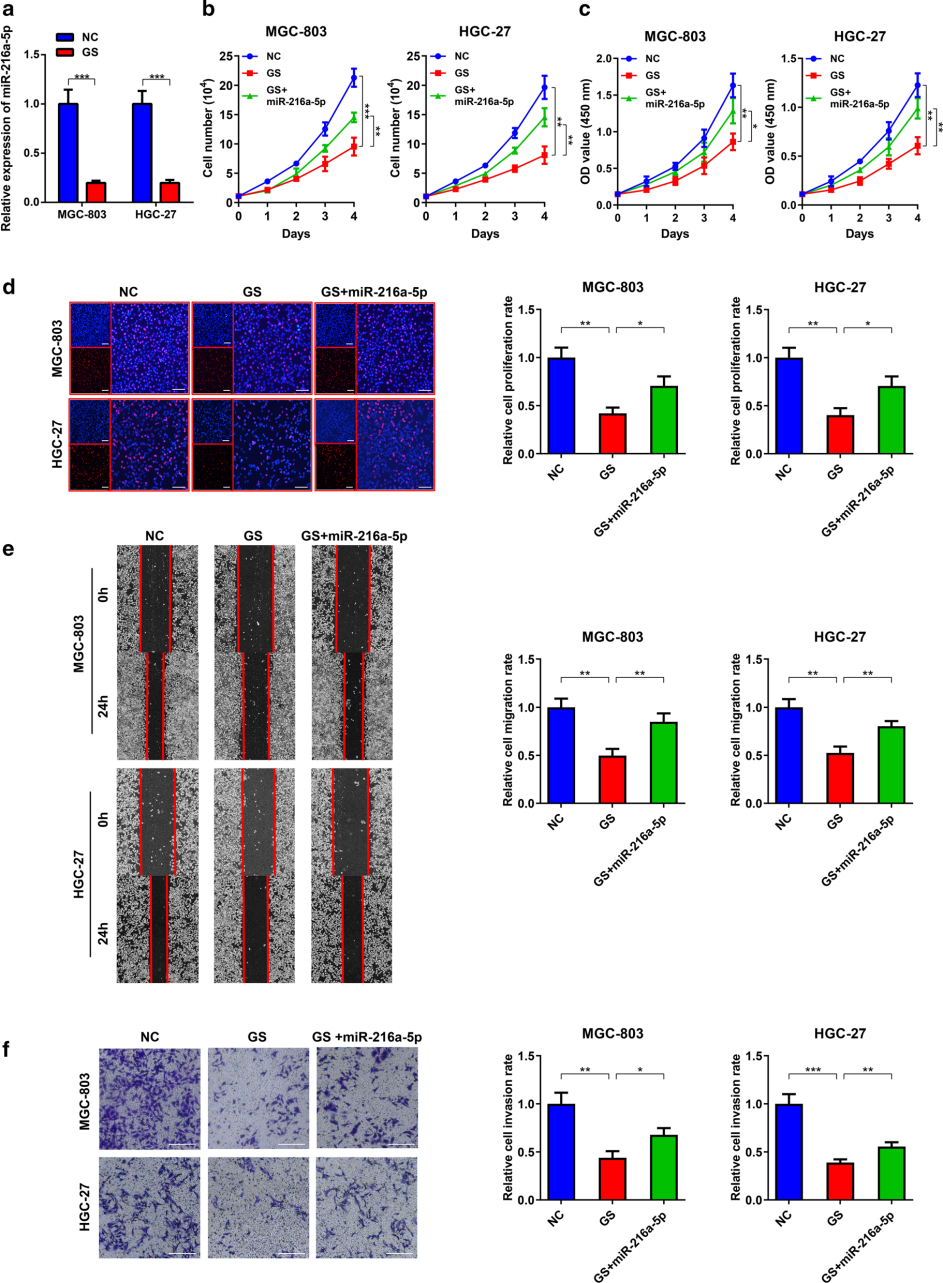
miR-216a-5p serves as an important mediator of FDFT1 upregulation induced by GS. **a** qRT-PCR analysis to show the relative expression of miR-216a-5p in MGC-803 and HGC-27 cells under the normal glucose condition or GS condition, respectively. **b** Cell counting assay to show proliferation of MGC-803 and HGC-27 cells transfected with negative control or miR-216a-5p mimics under normal glucose condition or GS condition, respectively. **c** CCK-8 assay to show proliferation of MGC-803 and HGC-27 cells as in (**b**). **d** EdU assay to show proliferation of MGC-803 cells and HGC-27 cells as in (**b**). Histograms are on the right. Scale bar: 100 mm. **e** Wound healing assay to show migration of MGC-803 and HGC-27 cells as in (**b**). Histograms are on the right. **f** Transwell assay to show invasion of MGC-803 and HGC-27 cells as in (**b**). Histograms are on the right. Scale bar: 100 mm. Data are presented as means ± SD. *P < 0.05, **P < 0.01, ***P < 0.001

It was speculated that GS could impair the miR-216a-5p effects based on their opposite influences on FDFT1 expression. The results showed that the cultivation under GS conditions reduced miR-216a-5p expression in MGC-803 and HGC-27 cells (Fig. 6A). Furthermore, in-vitro experiments proved that compared to the normal glucose condition, GS inhibited GC proliferation, migration, and invasion. Recovery of miR-216a-5p expression partially restored the malignant behaviors (Fig. 6B–F). These results suggested that low-glucose environments inhibited GC progression partially by suppression of miR-216a-5p expression.

### GS suppresses GC growth by targeting miR-216a-5p/FDFT1 axis in vivo

**Fig.7:**
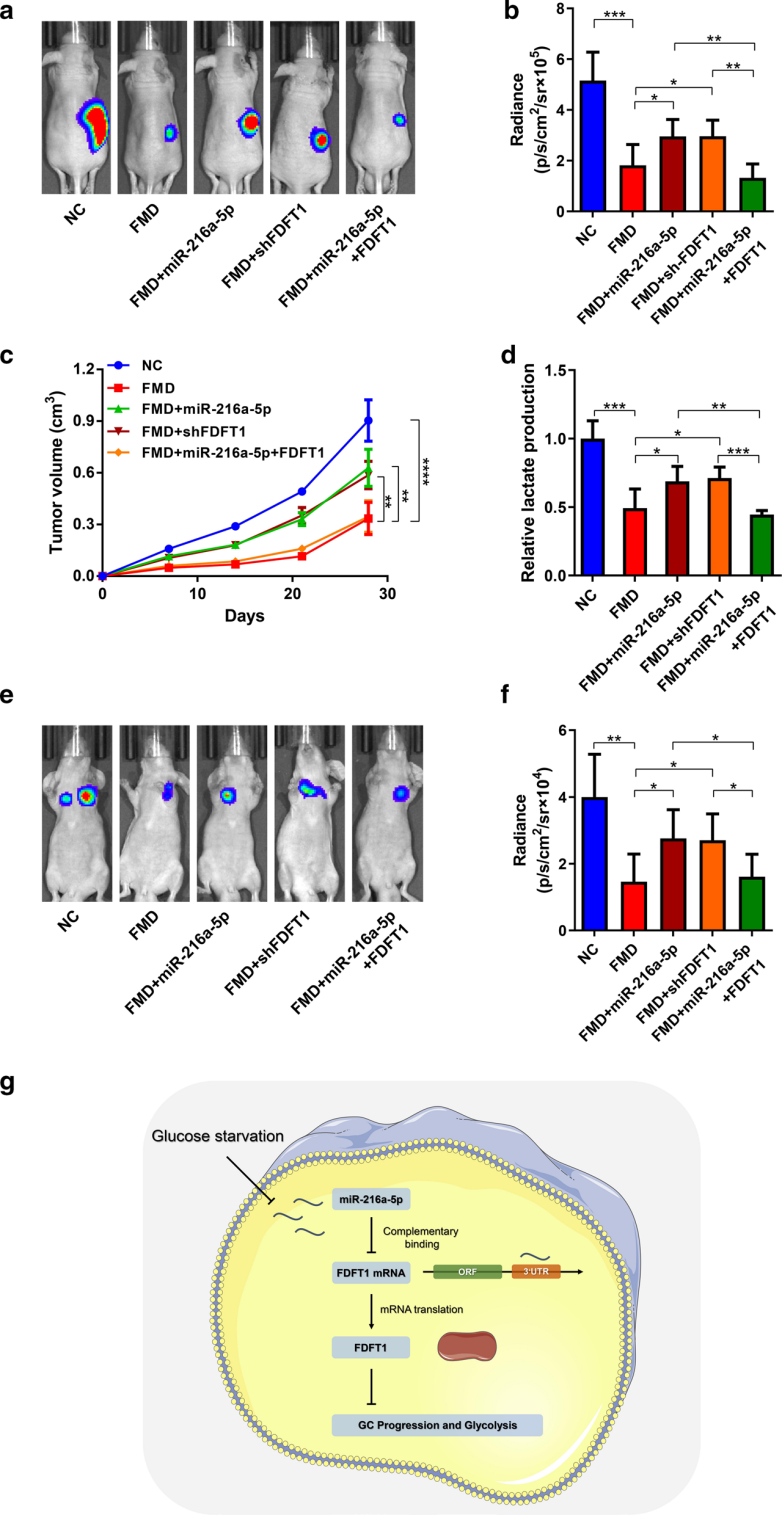
GS suppresses GC growth by targeting miR-216a-5p/FDFT1 axis in vivo. **a** Representative bioluminescence images at 30 days after subcutaneous injection of luc-MGC-803 cells with overexpression of vectors or FDFT1 and NC or miR-216a-5p mimics, respectively. Mice were fed by normal diet or FMD. **b** Luminescence signals in (**a**) represented by overlaid false-color images with the signal intensity. **c** Curves of tumor volumes as in (**a**) at the indicated time. **d** Lactate production was determined in the tumor tissues from (**a**). **e** Representative bioluminescence images at 30 days after tail vein injection of luc-MGC-803 cells with overexpression of vectors or FDFT1 and NC or miR-216a-5p mimics, respectively. Mice were fed by normal diet or fasting diet. **f** Luminescence signals in (**e**) represented by overlaid false-color images with the signal intensity. **g** Schematic illustration of miR-216a-5p/FDFT1 axis regulated by GS in this study. Data are presented as means ± SD. *P < 0.05, **P < 0.01, ***P < 0.001, ****P < 0.0001

To further validate the association between GS and miR-216a-5p/FDFT1 axis, we generated cell-derived xenografts by subcutaneous injection of MGC-803 cells. The luminescent intensity serve as an indicator of GC burden and the growth curve displayed the speeds of GC proliferation in vivo. Scheduled fasting was used to generate hypoglycemia and mimic GS. Compared to the control group, intermittent fasting diet effectively inhibited GC growth, which was antagonized by knockdown of FDFT1. Furthermore, miR-216a-5p overexpression promoted GC growth in FMD mice. Overexpression of FDFT1 restored the promotive effects of miR-216a-5p (Fig. 7A–C). Lactate acid production in subcutaneous tumors was determined and exhibited the similar tendency with tumor growth (Fig. 7D). For the detection of lung metastasis, tumors cells were injected into tail veins. After 30 days, FMD suppressed metastatic capability of GC cells. Upregulation of miR-216a-5p and FDFT knockdown could impair the anti-cancer effects of FMD. However, overexpression of FDFT1 could counteract the functions of miR-216a-5p (Fig. 7E, F). Our findings clarified the biological functions of miR-216a-5p/FDFT1 axis in GC suppression led by GS both in vitro and in vivo (Fig. 7G).

## Discussion

Glucose is the main energy source for cancer development. Numerous studies verified potential value of GS in cancer treatment and elucidated the underlying mechanisms. A great number of critical regulators were identified to participate in the GS. Baik et al. found that ZBP1 mediated necroptosis of breast cancer induced by GS [21]. RHOF also acts as a protector of hepatocellular carcinoma and promotes metabolic reprogramming under glucose deprivation conditions [22]. However, cancer cells tend to rob nutrition from the human body to adapt to their highly proliferative states. Patients with advanced and late stages cancer usually suffer from cachexia, unable to tolerate the side effects of a long-term fasting diet. It is urgent to elucidate the mechanisms by which GS counteracts cancer progression and develop novel therapeutic regimens combined with FMD.

FDFT1 determines the flow of farnesyl pyrophosphate to sterol or non-sterol metabolite pathways [23]. FDFT1 expression was reported under the rigorous control of SREBP1, a common regulatory factor in lipid metabolism [24]. Dysregulation of FDFT1 is associated with many diseases, such as hypoxic injury, nonalcoholic steatohepatitis, and chronic hepatitis C [25–27]. Its role in cancer progression is controversial. Aya et al. demonstrated that FDFT1 knockdown attenuated stemness of ovarian cancer [28]. However, Lu et al. found that it could inhibit proliferation of ovarian cancer [12]. Downregulation of FDFT1 expression also inhibited prostate cancer proliferation [29]. Its role in GC was unclear before our findings. In this study, we found that FDFT1 was overexpressed in GC and negatively correlated with some pathological characteristics, including pT stage, pTNM stage and cancer differentiation. High FDFT1 expression in GC tissues indicated better prognosis, especially for GC patients at stage III and IV. Bioinformatic analysis suggested that FDFT1 associates with GC progression and metabolism. Experiments proved that FDFT1 overexpression inhibited GC malignant behaviors, while knockdown of it enhanced the progression. Given that it was reported to be under the regulation of GS in colorectal cancer [11], we next verified this relationship in GC. Gain- and loss-of-function experiments showed that FDFT1 served as an important mediator of inhibitive effects induced by GS.

The miRNA regulatory networks participate in carcinogenesis and metastasis. Interference of them can effectively inhibit cancer progression [14, 30, 31]. For GC, the RNA-binding protein DDX18 promoted progression by contributing to miR-21 maturation and activation of the AKT signaling pathway [32]. miR-130b enhanced 5-FU resistance of GC cells by suppressing CMPK1 expression [33]. miR-216a-5p was also reported to promote malignancy of multiple tumors by sponging many targets [34–36]. To investigate upstream miRNAs of FDFT1 in GC, we employed five kinds of bioinformatic tools and four miRNAs were selected out. Further interaction experiments demonstrated that only miR-216-5p could attenuate FDFT1 expression. Specifically, miR-216a-5p bond to the FDFT1 mRNA 3’UTR to suppress its expression. The assays verified that miR-216a-5p/FDFT1 axis could regulate GC proliferation, migration, invasion, and glycolysis both in vitro and in vivo.

There are some studies concerning the mechanisms of GS-mediated alterations of miRNA networs. For instance, miR-451 was proved to regulate glioblastoma multiforme adaptation to GS [16]. To the best of our knowledge, there has been no research that focused on the glucose deprivation and downstream miRNA networks in GC before this work. Regarding that FDFT1 was simultaneously under the contradictory regulation of glucose deprivation and miR-216a-5p, it was speculated that GS elevates FDFT1 levels by decreasing miR-216a-5p expression in GC. Gain- and loss-of-function experiments verified that GS inhibited miR-216a-5p expression, rescuing the inhibitory effects on FDFT1 at the post-transcriptional level.

There are some limitations in this study. First, we only found that decreasing glucose supplement led to miR-216a-5p expression downregulation. The underlying mechanisms of this regulatory relationship needs further investigations. Second, GS/miR-216a-5p/FDFT1 axis was identified in GC. More experiments should be performed to clarify it in more types of cancer. Third, GC heterogeneity is an important factor that decreases efficacies of current drugs. The value of interfering miR-216a-5p/FDFT1 axis in GC treatment require more convincing proofs.

## Conclusions

In summary, our findings proved that FDFT1 served as a suppressor of GC progression and glycolysis. GS is a classical approach to limiting cancer growth, which inhibits malignancy by regulating the miR-216a-5p/FDFT1 axis. FDFT1 may become a prognostic factor and therapeutic target combined with fasting diet in the future.

## Supplementary Information

**Additional file 1: Fig. S1**. Correlation and enrichment analysis of FDFT1. a The heatmap to display the top 20 genes that most positively associated with FDFT1 expression and the top 20 genes that most negatively associated with FDFT1. b KEGG network to display top 10 pathways associated with FDFT1 expression. c Top 20 BP terms that were significantly enriched in GO analysis. d Top 20 CC terms that were significantly enriched in GO analysis. e Top 20 MF terms that were significantly enriched in GO analysis

**Additional file 2: Fig. S2.** The interference efficiencies of miRNA mimics. a, b qRT-PCR analysis to detect miRNA expression in cells transfected with NC and the corresponding miRNA mimics, including miR-216a-5p, miR-370-3p, miR-548c-3p and miR-607 mimics in MGC-803 and HGC-27 cells. Data are presented as means±SD. **P < 0.01, ***P < 0.001, ****P < 0.0001.

**Additional file 3: Fig. S3**. miR-216a-5p promotes proliferation, migration and invasion by targeting FDFT1 in GC cells. a Cell counting assay to show proliferation of MGC-803 and HGC-27 cells stably carrying lentivirus with vectors or FDFT1 overexpression plasmids and transfected with NC mimics or miR-216a-5p mimics, respectively. b CCK-8 assay to show proliferation of MGC-803 and HGC-27 cells as in (a). c EdU assay to show proliferation of MGC-803 cells and HGC-27 cells as in (a). Histograms are on the right. Scale bar: 100 mm. d Wound healing assay to show migration of MGC-803 cells and HGC-27 cells as in (a). Histograms are on the right. e Transwell assay to show invasion of MGC-803 cells and HGC-27 cells as in (a). Histograms are on the right. Scale bar: 100 mm. Data are presented as means±SD. *P < 0.05, **P < 0.01.

**Additional file 4**: **Table S1**. The sequences of oligo dT and primers used in this study.

## Data Availability

The datasets generated and/or analyzed during the current study are available from the corresponding author on reasonable request.

## Abbreviations

GC: Gastric cancer
FDFT1: Farnesyl-Diphosphate Farnesyltransferase 1
GS: Glucose starvation
3’UTR: 3’ Untranslated region
miRNA: MicroRNA
TCGA: The Cancer Genome Atlas
GO: Gene ontology
BP: Biological process
CC: Cellular component
MF: Molecular function
KEGG: Kyoto Encyclopedia of Genes and Genomes
CCK-8: Cell counting kit-8
EdU: 5-Ethynyl-2-deoxyuridine
qRT-PCR: Quantitative real-time PCR
WB: Western blot
OS: Overall survival
PFS: Progression-free survival
ECAR: Extracellular acidification rate
OCR: Oxygen consumption rate
FMD: Fasting mimic diet
OS: Overall survival
PFS: Progression-free survival
pT stage: Pathological T stage
pTNM stage: Pathological TNM stage
