# Supplementary figures and images for: Glucose starvation suppresses gastric cancer through targeting miR-216a-5p/Farnesyl-Diphosphate Farnesyltransferase 1 axis
Source: Cancer Cell Int. 2021 Dec 25;21:704. doi: 10.1186/s12935-021-02416-7 (PMC8710003; doi:10.1186/s12935-021-02416-7)

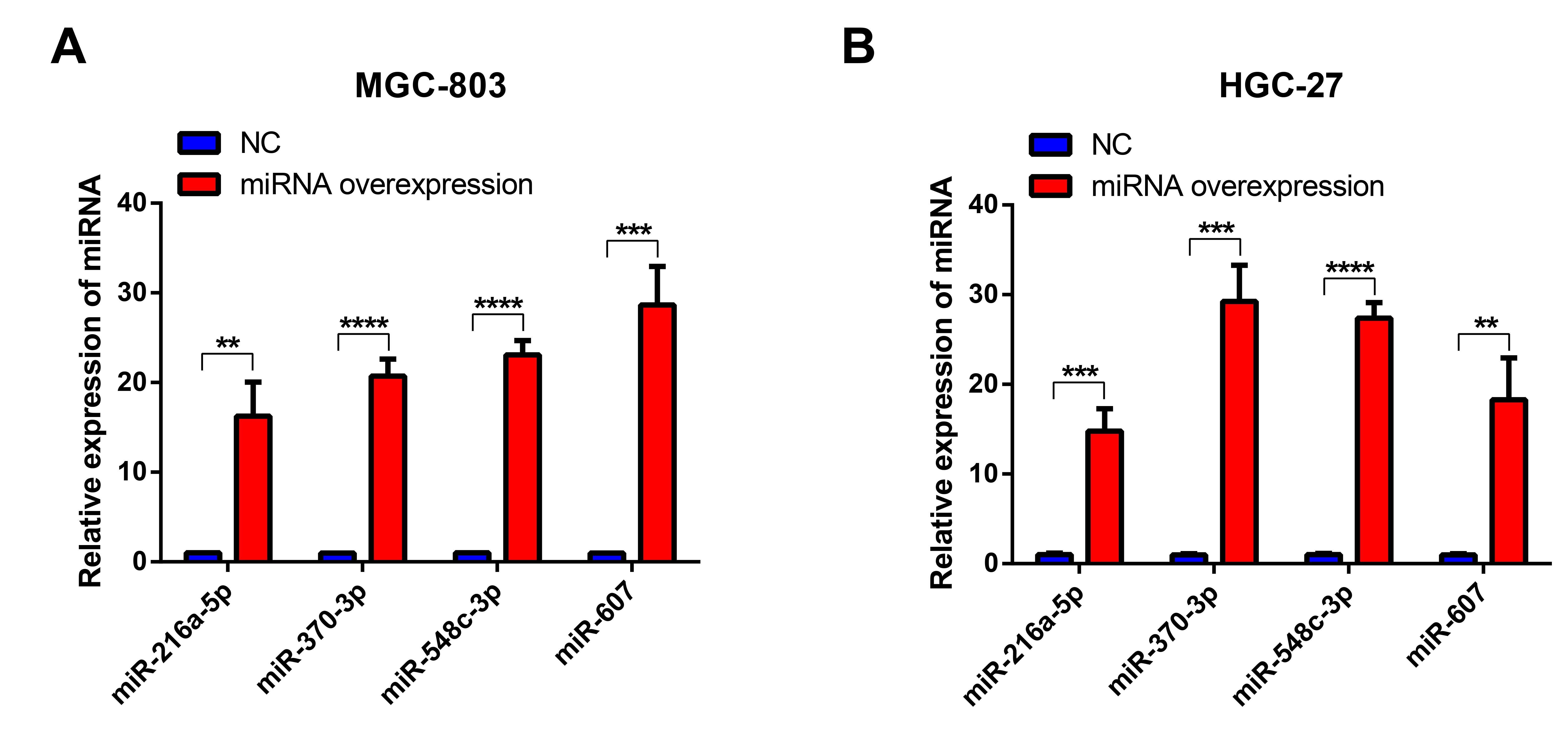

Supplement: Supplementary file 2 — Additional file 2: Fig. S2. The interference efficiencies of miRNA mimics. a, b qRT-PCR analysis to detect miRNA expression in cells transfected with NC and the corresponding miRNA mimics, including miR-216a-5p, miR-370-3p, miR-548c-3p and miR-607 mimics in MGC-803 and HGC-27 cells. Data are presented as means±SD. **P < 0.01, ***P < 0.001, ****P < 0.0001. [file 12935_2021_2416_MOESM2_ESM.jpg]
