# Supplementary material for: Glucose starvation suppresses gastric cancer through targeting miR-216a-5p/Farnesyl-Diphosphate Farnesyltransferase 1 axis
Source: Cancer Cell Int. 2021 Dec 25;21:704. doi: 10.1186/s12935-021-02416-7 (PMC8710003; doi:10.1186/s12935-021-02416-7)
Supplement: Supplementary file 4 — Additional file 4: Table S1. The sequences of oligo dT and primers used in this study. [file 12935_2021_2416_MOESM4_ESM.docx]

**Table S1 The sequences of oligo dT and primers used in this study.**

| Definition | Sequence (5’-3’) |
| --- | --- |
| miR-216a-5p oligo dT | GTCGTATCCAGTGCAGGGTCCGAGGTATTCGCACTGGATACGACTCACAG |
| mir-370-3p oligo dT | GTCGTATCCAGTGCAGGGTCCGAGGTATTCGCACTGGATACGACACCAGG |
| miR-548c-3p oligo dT | GTCGTATCCAGTGCAGGGTCCGAGGTATTCGCACTGGATACGACGCAAAA |
| miR-607 oligo dT | GTCGTATCCAGTGCAGGGTCCGAGGTATTCGCACTGGATACGACGTTATA |
| FDFT1 Forward | GGAAGACCAGCAAGGAGGAA |
| FDFT1 Reverse | ACTGCACGGCCAAGTCAATA |
| β-actin Forward | CATCCGCAAAGACCTGTACG |
| β-actin Reverse | CCTGCTTGCTGATCCACATC |
| miR-216a-5p Forward | TAATCTCAGCTGGCAA |
| miR-370-3p Forward | GCCTGCTGGGGTGGAA |
| miR-548c-3p Forward | CAAAAATCTCAATTAC |
| miR-607 Forward | GTTCAAATCCAGATC |
| Universal reverse primer of miRNAs | GTGCAGGGTCCGAGGT |
| U6 Forward | CTCGCTTCGGCAGCACA |
| U6 Reverse | AACGCTTCACGAATTTGCGT |
